# Clinical isolates of *Tritrichomonas foetus* in bulls in Wyoming, South Dakota and Montana, USA

**DOI:** 10.1186/s12917-020-2229-6

**Published:** 2020-01-10

**Authors:** Yinzhu Jin, Aifang Du, Chaoqun Yao

**Affiliations:** 1 Department of Veterinary Sciences, University of Wyoming, Laramie, WY 82070 USA; 2 Present Address: Division of Pharmacoepidemiology and Pharmacoeconomics, Department of Medicine, Brigham and Women’s Hospital and Harvard Medical School, Boston, MA 02115 USA; 3 Zhejiang Provincial Key Laboratory of Preventive Veterinary Medicine, Institute of Preventive Veterinary Medicine, College of Animal Sciences, Zhejiang University, Hangzhou, 310058 China; 4 Department of Biomedical Sciences and One Health Center for Zoonoses and Tropical Veterinary Medicine, Ross University School of Veterinary Medicine, P.O. Box 334, Basseterre, St. Kitts and Nevis

**Keywords:** *Tritrichomonas foetus*, Trichomonosis, rRNA gene, Cysteine protease, Cattle

## Abstract

**Background:**

Several *Tritrichomonas* species have been found in mammalian hosts. Among these trichomonads *T. foetus* is often found in the urogenital tract of cattle and the gastrointestinal tract of the domestic cat, resulting in sexually transmitted bovine trichomonosis and fecal-orally transmitted feline trichomonosis, respectively. The aims of the current study were to molecularly characterize clinical isolates of *T. foetus* in cattle populations in Wyoming, South Dakota, and Montana of the United States of America and to phylogenetically analyze *Tritrichomonas* species of mammalian hosts.

**Results:**

DNA sequencing of rRNA genes showed over 99% identity of the newly described isolates to other bovine isolates. Further, *T. foetus* isolates of various mammalian hosts originated in different geographic regions worldwide were clustered into two well-defined clades by phylogenetic analysis of rRNA and cysteine protease 2 genes. Clade I consisted of isolates originated from cattle, pig, and human whereas clade II contained isolates of cat and dog.

**Conclusion:**

It is concluded that all mammalian *Tritrichomonas* spp. apparently belong to *T. foetus*. Analysis of more sequences is warranted to support this conclusion.

## Background

*Tritrichomonas foetus* is a trichomonad protozoan of worldwide distribution in cattle populations in both developed and developing countries. It is often found in the urogenital tract of bovine hosts whose reproduction is mainly achieved by natural service of coitus. Interestingly, the protozoan is more effectively transmitted from an infected male to a susceptible female than from a cow to a bull. 95% of susceptible nulliparous cows became infected after a single mating with a *T. foetus*-positive bull [16]. In contrast, it took three to six times of mating for *T. foetus*-negative Hereford bulls of age 4–6 years to become *T. foetus* positive when they were allowed to mate with *T. foetus*-positive heifers. Furthermore, one three-year old bull became positive only after nine times of mating whereas the other still remained negative afterwards [2]. A bull usually becomes a lifetime carrier once being infected whereas a cow naturally clears infection in a few months postinfection [26]. Occasionally a cow remains *T. foetus* positive for a longer period of time. For example, she was still positive 22 months after infection or 9 weeks after her having given birth to a calf post a normal pregnancy [1, 17].

Bovine trichomonosis is endemic in the United States of America (USA) especially in the states west of the Mississippi River [28]. The control and eradication method of bovine trichomonosis that has been proven most effective is artificial insemination (AI). AI has been being practiced throughout the European Union (EU) where *T. foetus* infection in cattle is practically nonexistent. For example, only two cases of bovine trichomonosis were found in the United Kingdom (UK) in 20 years [22]. We have recommended AI as the top choice followed by culling *T. foetus*-positive bulls to curtail its endemic in the USA [8, 26]. However, not all US cattle farmers are practicing AI or are willing to perform AI due to facilities or cost effectiveness, or various constraints of their operations. In a questionnaire study carried by us in Wyoming in the Spring 2011, only 2.1% of Wyoming cattle producers used AI and another 36.7% would consider AI in the future [8]. The current measure of testing all bulls for *T. foetus* and culling *T. foetus*-positive ones had led to a gradual and steady decrease in bovine prevalence in Wyoming from 2.69% in 1999, the year immediately prior to regulation enforcement, to 0.21% in year 2010 [28]. The control method appeared effective, but there was still a long way to control and eventually to eradicate bovine trichomonosis in the USA.

In addition to cattle, *T. foetus* also infects the domestic cat. Different from that in cattle, the predilection site of feline infection is the gastrointestinal tract which may lead to diarrhea, often a chronic one [30]. Nevertheless, a different species, i.e., *T. blagburni,* had been proposed for the protozoan in the feline host [24], although the name had not been widely accepted yet. In addition, *T. suis* in swine host and *T. foetus* in cattle were considered synonymous [10, 20]. *T. foetus* was suggested as a valid name for the species due to its wide use for being the etiological agent of bovine trichomonosis even though *T. suis* was a senior synonym to *T. foetus* [18]. Furthermore, *T. foetus* bovine isolate had been occasionally reported in human [25, 29]. There is paucity and confusion about *Tritrichomonas* species infecting these mammalian hosts. The aims of current study were to molecularly characterize *T. foetus* in the bovine host in the northwest region of USA and to phylogenetically analyze *Tritrichomonas* species among these various mammalian hosts using molecular data deposited in the GenBank.

## Results

During the 2 years of sampling time, one positive sample was submitted from a herd in Blaine County, Montana and two from Butte County, South Dakota. There were 34 positive batches of samples from eight Wyoming counties, i.e., Carbon, Crook, Fremont, Hot Spring, Lincoln, Sweetwater, Unita, and Washakie (Table 1). Six batches of the 37 samples were submitted in frozen for PCR diagnosis. Among the 31 culture positive samples, five failed to multiply during passing in Diamond’s media, resulting in a failure rate of 16.1%.

**Table 1:** *Tritrichomonas foetus* isolates included in this study

Isolates	County, State	Sample tested	No positive^+^	Sampling date	Cultured	Sequenced
SD1	Butte, SD	4	1^+^	1/18/2011	+	+
SD2	Butte, SD	21	1	4/12/2011	+	+
WY1	Unita, WY	1	1^+^	1/24/2011	+	+
WY2	Unita, WY	1	1	2/18/2011	+	+
	Fremont	2	1	2/22/2011	–	–
WY3	Unita, WY	1	1^+^	3/28/2011	+	+
WY4–6	Unita, WY	4	3^+^	4/8/2011	+	+
	Carbon, WY	48	2	4/15/2011	–	–
WY7	Hot Spring, WY	73	1	4/20/2011	+	+
WY8.1–8.2	Carbon, WY	18	7^+#^	5/12/2011	+	+
WY9	Crook	11	1^+^	5/13/2011	+	+
	Hot Spring, WY	25	2	5/23/2011	–	–
WY10	Hot spring, WY	32	1	6/22/2011	+	+
WY12–13	Unita, WY	2	2^+^	12/31/2011	+	+
WY11	Lincoln, WY	39	1^+^	1/5/2012	+	+
WY14	Unita, WY	1	1^+^	1/14/2012	+	–
WY15	Unita, WY	51	1^+^	1/30/2012	+	–
WY16	Sweetwater, WY	1	1^+^	1/28/2012	–	–
WY17–18	Lincoln, WY	2	2^+^	2/7/2012	+	–
WY19.1–19.9	Lincoln, WY	109	13*	2/15/2012	–	–
WY20.1–20.2	Lincoln, WY	30	2^+^	3/8/2012	+	–
WY21	Fremont, WY	3	1^+^	4/3/2012	+	–
WY22.1–22.2	Washakie, WY	14	3	10/17/2012	+	–
Wy23.1–23.2	Washakie, WY	80	2	11/29/2012	+	–
WY24.1–24.2	Washakie, WY	36	2	12/4/2012	+	–
WY25	Washakie, WY	95	1^+^	12/11/2012	+	–
	Washakie, WY	34	1	12/11/2012	–	–
	Unita, WY	5	2*	12/19/2012	–	–
	unknown, WY	6	2*	1/3/2013	–	–
	Sweetwater, WY	12	4*	1/14/2013	–	–
WY26.1–26.6	Washakie, WY	62	6	1/10/2013	+	–
	Hot Spring, WY	73	1	1/16/2013	+	–
	Sweetwater, WY	8	1*	1/19/2013	–	–
MT 1	Blaine, MT	55	2*	1/7/2013	–	–

^+^: culture positive samples also confirmed by PCR

^#^: Only two of seven sample were available for culture

*: Frozen samples were submitted for PCR only

The new primers targeted a 608 bp fragment that enclosed the entire 347 bp sequence amplified by the primers TFR3 and TFR4 being used in WSVL in diagnosing *T. foetus* infections [28]. In total, two and 14 isolates in South Dakota and Wyoming, respectively, were PCR amplified using the new primers, each yielded a single amplicon of the expected size. After DNA sequencing all PCR products had identical sequences. Two representative sequences minus primers, i.e., WY10 and SD1, were submitted to the GenBank. The accession numbers were MK250821 and MK250822, respectively. The new sequences borne 99.82% identity to M81842 (563/564) and 99.82% to AF466749 (540/541) of *T. foetus*, and 92.13% to JF927156 (527/572) of a *Tritrichomonas* sp. of an avian host by BLAST search.

**Fig. 1:**
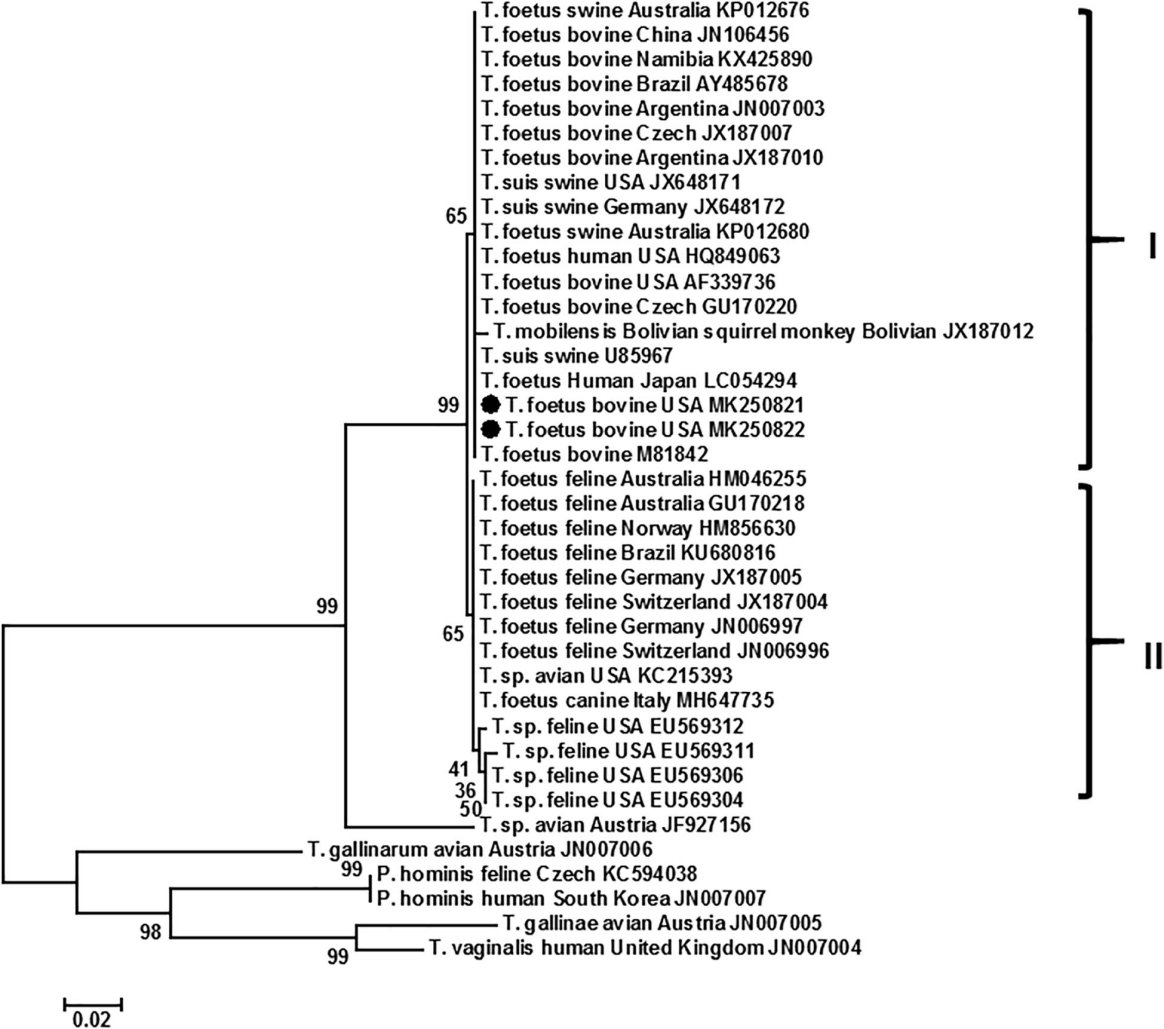
Phylogenetic tree of trichomonad species using rRNA genes. The two new DNA sequences (marked by •) of *T. foetus* of bovine host along with related entries in GenBank were analyzed by Neighbor-Jointing method of MEGA under a default setting of p-distance model, uniform rates and complete depletion with 1000 bootstrap replications. Numbers at the horizontal lines represent percentage of replicates of 1000 repeats. Scale bar indicates nucleotide substitutions per site. Two clades (I-II) of *T. foetus* are marked. Each entry is in the order of parasite name, host, country of the parasite origination if known, and GenBank accession number. Maximum Likelihood method in the default setting yielded identical results (Not shown)

We then performed a phylogenetic analysis of the two new rRNA sequences along with the entries of corresponding sequences of various trichomonads deposited in the GenBank. As showed in Fig. 1, trichomonad species such as *Trichomonas vaginalis*, *T. gallinae*, *Pentatrichomonas hominis* and *Tetratrichomonas gallinarum* were all well separated into branches of species individually and distanced from the cluster of *Tritrichomonas* spp., which included two species. A yet to be named *Tritrichomonas* sp. isolated from an avian host in Austria was closely yet distinctly separated from *T. foetus*. The latter were clearly divided into two closely-related clades. Clade I contained isolates originated in bovine, swine, squirrel monkey and human hosts in the geographical regions of North America, South America, Europe, Asia, Africa and Australia/Oceania. Clade II contained isolates of feline, canine and avian hosts in North America, South America, Europe and Australia/Oceania (Fig. 1).

**Fig. 2:**
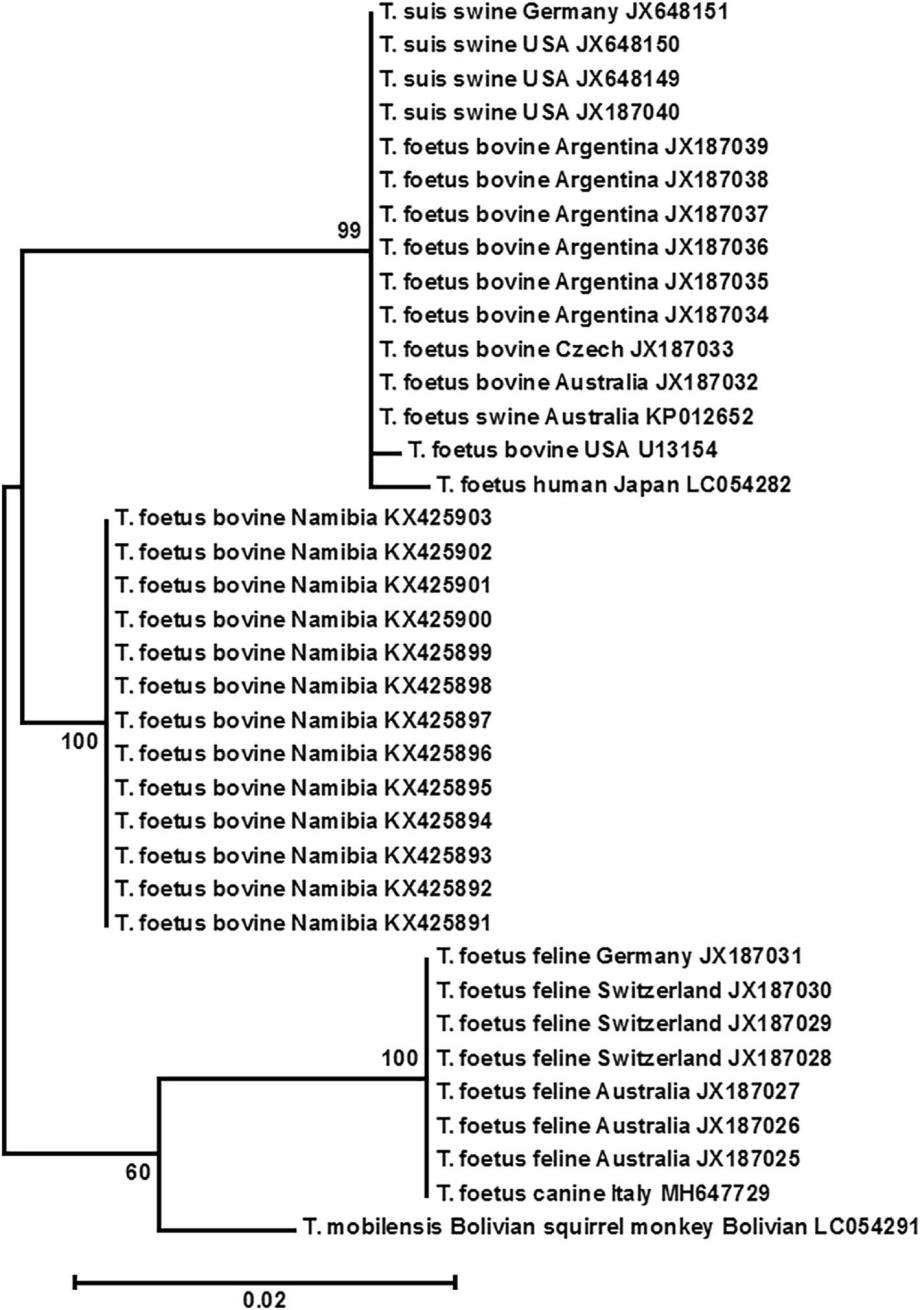
Phylogenetic tree of *Tritrichomonas* species using cysteine protease 2 DNA sequence. Phylogenetic analysis was performed on 37 entries available in GenBank using Maximum Likelihood method in the default setting of MEGA. Same conditions as those in Fig. 1 were used. Neighbor-Jointing method yielded similar results (Not shown)

Phylogenetic analysis of *CP2* DNA sequences was also performed. Unfortunately, the phylogenetic tree was unrooted in this case due to lack of an outgroup of homologous sequences from other trichomonad species. Nevertheless, similar results as the rRNA analysis were obtained. Again, clade I included bovine, swine and human isolates in North America, South America, Europe, Asia, Africa and Australia/Oceania. Clade II consisted of feline and canine isolates of Europe and Australia. An isolate originated from Bolivian squirrel monkey was closely aligned with Clade II (Fig. 2).

## Discussion

*Tritrichomonas foetus* is the causative agent of bovine trichomonosis, a widespread sexually transmitted disease of cattle in many geographical regions worldwide. The disease has been reported in six continents including North America, South America, Europe, Asia, Africa and Australia/Oceania. Although being eradicated in many EU countries the disease still exists in some foci where AI is not carried out. One example is Asturiana de la Montana, Northern Spain, where the prevalence of the parasite in bulls was 32% in 2012 [4, 11, 13]. Interestingly, the disease was absent in cattle on St. Kitts, West Indies even though natural service of mating was the main reproductive method of the cattle populations on the island [3]. The disease was endemic in many US states including Alabama, California, Colorado, Florida, Idaho, Kansas, Missouri, Montana, New Mexico, Nebraska, Nevada, Oklahoma, South Dakota, Utah and Wyoming [28]. In an effort to control the disease many US states including all states west of the Mississippi River except Minnesota along with Mississippi, Alabama, Georgia, Tennessee and Hawaii have state rules and regulations on bovine trichomonosis. In this study available samples of positive *T. foetus* bulls diagnosed by WSVL between January 2011 and January 2013 were included, which covered samples from South Dakota, Montana and Wyoming. One shortcoming of the study is that prevalence of *T. foetus* could not be calculated because the study did not cover all herds in these states. Nevertheless, one of our earlier studies showed that annual prevalence of Wyoming bulls from year 2000 to 2010 was between 0.21 and 1.69%. Furthermore, numbers of positive bulls between 2006 to 2010 were annually recorded as 57, 50, 45, 98 and 17, respectively [28]. This current study showed positive bulls in Wyoming in 2011 and 2012 were 23 and 47, respectively. Collectively, our data showed the least numbers of positive bulls in years 2010 and 2011 over a decade period of time since 2000 when state bovine trichomonosis rules started to be enforced. However, number of the positive bulls was more than doubled in the year 2012 compared to the two previous years, which might be caused by complacency of some cattle farmers and should raise some concerns. A well-designed epidemiological study needs to be carried out to address what caused this recurrence if the trend continues.

Additionally, *T. foetus* had been routinely found in the domestic cat in 21 countries in North America, South America, Europe, Asia and Australia/Oceania [30, 31]. However, it was not detected in the cat populations on St. Kitts, West Indies by culturing and PCR [31]. The feline strain differs from the bovine strain by one base pair in the ITS-2 sequence [25, 29]. In addition, there were some differences in other genes. Majority of these differences were less than 1% except cysteine protease 2 and 6, which were 3.3 and 1.9%, respectively [18, 29]. In addition, other *Tritrichomonas* spp. had been described in various mammals. One example is *T. suis* that is commonly found in nasal cavity, stomach, cecum, and colon of swine hosts as a commensal, and molecular data had suggested this species is a synonym of *T. foetus* [19, 20]. Sequence analysis of multiple genes also concluded that *T. suis* was synonymous to the bovine isolate of *T. foetus*. The bovine, feline and swine isolates should be given the name of *T. foetus* [18]. Another example is *T. mobilensis* of Bolivian squirrel monkeys, which was similar to *T. foetus* even at the ultrastructural level [12]. Our phylogenetic analysis on rRNA and CP2 DNA sequences of representative isolates around the world had clearly showed two very close clades of *T. foetus* (Figs. 1 and 2). Clade I included bovine isolates including the two new ones from South Dakota and Wyoming, swine isolates, and human isolates. Clade II included feline and canine isolates. The Bolivian squirrel monkey isolate was inconsistent. It was in clade I and II in rRNA and CP2 analysis, respectively (Figs. 1 and 2). The avian isolate was in the Pacific Coast band-tailed pigeon (*Patagioenas fasciata monilis*) from California and was considered due to coprophegia of infected cat feces by the pigeon [5]. The domestic dog had been infrequently found infected with *T. foetus* in USA, China and Italy. The prevalence was 0.6% (2/315) in Anhui Province and Zhejiang Province, China among the dogs visiting veterinary clinics with or without diarrhea and 2.6% in dogs with diarrhea in USA [9, 23]. In a survey of shelter dogs in Italy from April to September 2017, 1.7% (1/59) dogs were found positive with *T. foetus* “feline isolate” by microscopy, PCR and DNA sequencing [7]. In a survey of 245 kennel puppies with or without diarrhea in France in 2009, 15.8% (34/215) were positive for fecal trichomonads by microscopy. Nevertheless, PCR and DNA sequencing showed 26 of the 34 samples were *P. hominis* positive whereas none were positive for *T. foetus* [6]. It is very likely that *T. foetus* feline isolate may be a parasite of canine host that results in diarrhea in infected dogs. However, this needs to be further confirmed. Collectively, *Tritrichomonas* spp. in mammalian hosts including cattle, cat, pig, dog, human and squirrel monkey are all considered as *T. foetus* unless otherwise proven with new molecular data in the future. Nevertheless, a *Tritrichomonas* sp. found in the intestine of a common quail (*Coturnix coturnix*) was distantly clustered to the two clades of *T. foetus* (Fig. 1). The rRNA sequences showed approximately 95% identity to those of *T. foetus* [14]. Whether this species is the same one as the previously described *T. gigantica* in the cecum of the same species of bird in India [15] needs to be determined by molecular studies.

## Conclusion

Clinic isolates of *T. foetus* in cattle populations in South Dakota, Montana, and Wyoming were molecularly characterized by PCR and DNA sequencing of rRNA sequences. Phylogenetic analysis on rRNA and CP2 DNA sequences showed two clades of *T. foetus*. Clade I consisted of these new isolates along with other bovine isolates, swine isolates, and human isolate whereas clade II contained feline and canine isolates. All isolates harbored by these mammals were *T. foetus*.

## Methods

### Clinical isolates

Between January 2011 and January 2013 preputial samples from bulls collected at cattle farms and subsequently submitted to the Wyoming State Veterinary Laboratory (WSVL) by various individual veterinarians were included in this study. All bulls in reproduction were required an annual *T. foetus* test by state rules. WSVL employed both culturing in Diamond’s medium and conventional gel-PCR for clinical diagnosis of *T. foetus* infection in cattle [28]. Cultures were routinely discarded and destroyed according to SOP of WSVL after a diagnosis was made. However, all positive cultures were saved and passed on to a research laboratory for the current study followed diagnosis between January 2011 and January 2013. No identities of farmers, nor the tag numbers of *T. foetus* positive cattle were revealed. Consequently, a waiver was granted for the current study by the Institutional Animal Care and Use Committee (IACUC) of the University of Wyoming.

### Cell culture

The original clinical cultures were transferred from WSVL to a research lab in a cooler with its temperature being maintained at 37 °C with warm water. The transferring between buildings on campus took a maximum of 30 min. They were cultured immediately in Diamond’s medium as previously described [27]. Briefly, the original clinical cultures were diluted in three to four 50 ml conical tubes filled with 50 ml Diamond’s medium at a starting cell density of 1 × 10^4^ cells/ml and were incubated vertically in 37 °C without agitation. Cell growth was monitored daily using a hemocytometer, and cells were harvested at day 3–4 of culture when they reached approximately 5 × 10^6^ cells/ml by centrifugation at 800×*g* for 10 min at 4 °C. Cell pellets were stored in -20 °C after being washed twice in Hank’s Balanced Salt Solution (HBSS, ThermalFisher Scientific, Carlsbad, CA) by same centrifugation. For all isolates included in the current study only the first passaged cells were used in order to minimize biased selection of population of *T. foetus* cells that favored the cultural conditions.

### PCR and DNA sequencing

Cell pellets were thawed at room temperature and were lysed at 37 °C for 1 h in 1.0% SDS supplemented with 0.1 mg/ml protease K. DNA was extracted with phenol/chloroform/isoamyl alcohol mix in a ratio of 25:24:1 (Sigma, St Louis, MO) after being treated with 0.1 mg/ml RNAse at 37 °C for 10 min, and was quantified and qualified by spectrophotometry (NanoDrop, ThermalFisher Scientific). PCR primers were designed from the sequence of rRNA genes of *T. foetus* bovine isolate (GenBank accession number: M81842). The primers (forward 5′-CCCTTGTAAATGCGTGTCAACAG-3′; reverse 5′-CGAACTCTACTCTCTTCGGTCGCACT-3′) were synthesized by IDT (Coralville, IA). The targeted DNA sequence of 608 bp included partial small subunit rRNA, internal transcribed spacer (ITS) 1, 5S rRNA, ITS 2, and partial large subunit rRNA. A thermal cycle consisting of initial 95 °C 5 min, 35 cycles of 95 °C 30s, 64 °C 30s, and 72 °C 1 min followed by 72 °C 10 min was carried out in a Veriti™ 96-Well Thermal Cycler (ThermalFisher Scientific) for hot start PCR. PCR products were revealed on 1.2% agarose gel with TrackIt™ 100 DNA ladder (Invitrogen) and were purified afterwards using QIAquick PCR Purification Kit (QIAGEN, Germantown, MD). Purified PCR products were directly sequenced in both directions using 25 ng DNA and one PCR primer in the in-house sequencing facility of the University of Wyoming (Nucleic Acid Exploration Facility). Occasionally ambiguous nucleotides were manually read out from sequence chromatograph. A unanimous sequence was generated for each DNA sample using sequences of both directions.

### Phylogenetic analysis of rRNA and cysteine protease (CP) 2 genes

Keywords *Tritrichomonas* and rRNA were used to search nucleotide database of the National Center for biotechnology information (NCBI), which yielded 255 entries (https://www.ncbi.nlm.nih.gov/nuccore/?cmd=HistorySearch&querykey=7, accessed December 10, 2018). All entries that contained ITS sequences were manually downloaded and a phylogenetic analysis was performed along with the two new sequences generated in the current study and deposited into GenBank using MEGA software (version 5.2) [21]. Representative entries were included in the phylogenetic analysis if multiple sequences were originated from same host and in the same origin of country to avoid overwhelming a phylogenetic tree with too many entries. Both neighbor-jointing and maximum likelihood methods were performed under default conditions of the MEGA software

Similarly keywords *Tritrichomonas* Protease and CP2 were used to search NCBI database Resulting in 36 entries (https://www.ncbi.nlm.nih.gov/nuccore?term=((Tritrichomonas)%20AND%20protease)%20AND%20CP2 Accessed January 17 2019). An additional entry was added by BLAST. Phylogenetic analysis was performed using neighbor-jointing and maximum likelihood methods under default conditions of the MEGA software.

## Data Availability

All data generated or analyzed during this study are included in this published article. DNA sequences are submitted to GenBank with accession numbers of MK250821 and MK250822.

## Abbreviations

AI: Artificial insemination
CP: Cysteine protease
EU: European Union
HBSS: Hank’s Balanced Salt Solution
ITS: Internal transcribed spacer
UK: United Kingdom
USA: United States of America
WSVL: Wyoming State Veterinary Laboratory

## References

[1] Alexander GI (1953). An outbreak of bovine trichomoniasis in Queensland and its control. Aus Vet J.

[2] Clark BL, Parsonson IM, Dufty JH (1974). Letter: infection of bulls with *Tritrichomonas foetus* through mating with infected heifers. Aust Vet J.

[3] Coker KE, Lim JJ, Schleisman RL, Vosloo C, French HM, Samper JC, Callanan JJ, Gilbert RO, Sithole F, Yao C, Chapwanya A (2018). Freedom from *Tritrichomonas foetus* infection in cattle in St. Kitts. Trop Anim Health Prod.

[4] Collantes-Fernández E, Mendoza-Ibarra JA, Pedraza-Díaz S, Rojo-Montejo S, Navarro-Lozano V, Sánchez-Sánchez R, Ruiz-Santa-Quiteria JA, Ortega-Mora LM, Osoro K (2014). Efficacy of a control program for bovine trichomonosis based on testing and culling infected bulls in beef cattle managed under mountain pastoral systems of northern Spain. Vet J.

[5] Girard YA, Rogers KH, Woods LW, Chouicha N, Miller WA, Johnson CK (2014). Dual-pathogen etiology of avian trichomonosis in a declining band-tailed pigeon population. Infect Genet Evol.

[6] Grellet A, Brunopolack, Feugier A, Boucraut-Baralon C, Grandjean D, Vandewynckel L, Cian A, Meloni D, Viscogliosi E (2013). Prevalence, risk factors of infection and molecular characterization of trichomonads in puppies from French breeding kennels. Vet Parasitol.

[7] Iatta Roberta, Buonfrate Dora, Paradies Paola, Cavalera Maria Alfonsa, Capogna Antonio, Iarussi Fabrizio, Šlapeta Jan, Giorli Giovanni, Trerotoli Paolo, Bisoffi Zeno, Otranto Domenico (2018). Occurrence, diagnosis and follow-up of canine strongyloidiosis in naturally infected shelter dogs. Parasitology.

[8] Jin Y, Schumaker B, Logan J, Yao C (2014). Risk factors associated with bovine trichomoniasis in beef cattle identified by a questionnaire. J Med Microbiol.

[9] Li W-C, Wang K, Zhang W, Wu J, Gu Y-F, Zhang X-C (2016). Prevalence and molecular characterization of intestinal trichomonads in pet dogs in East China. Korean J Parasitol.

[10] Lun ZR, Chen XG, Zhu XQ, Li XR, Xie MQ (2005). Are *Tritrichomonas foetus* and *Tritrichomonas suis* synonyms?. Trends Parasitol.

[11] Mendoza-Ibarra JA, Pedraza-Diaz S, Garcia-Pena FJ, Rojo-Montejo S, Ruiz-Santa-Quiteria JA, San Miguel-Ibanez E, Navarro-Lozano V, Ortega-Mora LM, Osoro K, Collantes-Fernandez E (2012). High prevalence of *Tritrichomonas foetus* infection in Asturiana de la Montana beef cattle kept in extensive conditions in northern Spain. Vet J.

[12] Midlej V, Pereira-Neves A, Kist LW, Bogo MR, Benchimol M (2011). Ultrastructural features of *Tritrichomonas mobilensis* and comparison with *Tritrichomonas foetus*. Vet Parasitol.

[13] Miro G, Hernandez L, Montoya A, Arranz-Solis D, Dado D, Rojo-Montejo S, Mendoza-Ibarra JA, Ortega-Mora LM, Pedraza-Diaz S (2011). First description of naturally acquired *Tritrichomonas foetus* infection in a Persian cattery in Spain. Parasitol Res.

[14] Mostegl MM, Richter B, Nedorost N, Maderner A, Dinhopl N, Kubber-Heiss A, Weissenbock H (2012). Identification of a putatively novel trichomonad species in the intestine of a common quail (Coturnix coturnix). Vet Parasitol.

[15] Navarathnam ES (1970). A new species of *Tritrichomonas* from the caecum of the bird *Coturnix coturnix* Linneaus. Riv Parassitol.

[16] Parsonson IM, Clark BL, Dufty JH (1976). Early pathogenesis and pathology of *Tritrichomonas foetus* infection in virgin heifers. J Comp Pathol.

[17] Skirrow S (1987). Identification of trichomonad-carrier cows. J Am Vet Med Assoc.

[18] Slapeta J, Muller N, Stack CM, Walker G, Lew-Tabor A, Tachezy J, Frey CF (2012). Comparative analysis of *Tritrichomonas foetus* (Riedmuller, 1928) cat genotype, *T. foetus* (Riedmuller, 1928) cattle genotype and *Tritrichomonas suis* (Davaine, 1875) at 10 DNA loci. Int J Parasitol.

[19] Sun Z, Stack C, Slapeta J (2012). Sequence differences in the diagnostic region of the cysteine protease 8 gene of *Tritrichomonas foetus* parasites of cats and cattle. Vet Parasitol.

[20] Tachezy J, Tachezy R, Hampl V, Sedinova M, Vanacova S, Vrlik M, Van Ranst M, Flegr J, Kuldaa J (2002). Cattle pathogen *Tritrichomonas foetus* (Riedmuller, 1928) and pig commensal *Tritrichomonas suis* (Gruby & Delafond, 1843) belong to the same species. J Eukaryot Microbiol.

[21] Tamura K, Peterson D, Peterson N, Stecher G, Nei M, Kumar S (2011). MEGA5: molecular evolutionary genetics analysis using maximum likelihood, evolutionary distance, and maximum parsimony methods. Mol Biol Evol.

[22] Taylor MA, Marshall RN, Stack M (1994). Morphological differentiation of Tritrichomonas foetus from other protozoa of the bovine reproductive tract. Br Vet J.

[23] Tolbert MK, Leutenegger CM, Lobetti R, Birrell J, Gookin JL (2012). Species identification of trichomonads and associated coinfections in dogs with diarrhea and suspected trichomonosis. Vet Parasitol.

[24] Walden HS, Dykstra C, Dillon A, Rodning S, Givens D, Bird R, Newton J, Lindsay D (2013). A new species of Tritrichomonas (Sarcomastigophora: Trichomonida) from the domestic cat (Felis catus). Parasitol Res.

[25] Yao C (2012). Opportunistic human infections caused by tritrichomonas species: a mini-review. Clin Microbiolo Newsl.

[26] Yao C (2013). Diagnosis of *Tritrichomonas foetus*-infected bulls, an ultimate approach to eradicate bovine trichomoniasis in US cattle?. J Med Microbiol.

[27] Yao C. Tritrichomonas foetus infections in female beef cattle with abortion in Wyoming. USA JMM Case Reports. 2015;2(2):1–5. 10.1099/jmmcr.0.000028.

[28] Yao C, Bardsley KD, Litzman EA, Hall ML, Davidson MR (2011). *Tritrichomonas foetus* infection in beef bull populations in Wyoming. J Bacteriol Parasitol.

[29] Yao C, Ketzis JK (2018). Aberrant and accidental trichomonad flagellate infections: rare or underdiagnosed?. Trans R Soc Trop Med Hyg.

[30] Yao C, Koster L (2015). Tritrichomonas foetus infection, a cause of chronic diarrhea in the domestic cat. Vet Res.

[31] Yao C, Köster L, Halper B, Dundas J, Nair R (2018). Failure to detect *Tritrichomonas foetus* in a cross-sectional survey in the populations of feral cats and owned outpatient cats on St Kitts, West Indies. JFMS Open Rep.

